# The genome sequence of the Turnip Sawfly, *Athalia rosae* (Linnaeus, 1758)

**DOI:** 10.12688/wellcomeopenres.18993.1

**Published:** 2023-02-17

**Authors:** Liam M. Crowley, Gavin R. Broad, University of Oxford and Wytham Woods Genome Acquisition Lab, Natural History Museum Genome Acquisition Lab, Darwin Tree of Life Barcoding collective, Wellcome Sanger Institute Tree of Life programme, Wellcome Sanger Institute Scientific Operations: DNA Pipelines collective, Tree of Life Core Informatics collective, Andrew Green, Darwin Tree of Life Consortium

**Affiliations:** 1 University of Oxford, Oxford, UK; 2 Natural History Museum, London, UK; 3 Sawfly Recording Scheme, Bedford, Bedfordshire, UK

**Keywords:** Athalia rosae, Turnip Sawfly, genome sequence, chromosomal, Hymenoptera

## Abstract

We present a genome assembly from an individual female *Athalia rosae* (the Turnip Sawfly; Arhropoda; Insecta; Hymenoptera; Athaliidae). The genome sequence is 172 megabases in span. Most of the assembly is scaffolded into eight chromosomal pseudomolecules. The mitochondrial genome has also been assembled and is 16.3 kilobases in length. Gene annotation of this assembly on Ensembl identified 11,393 protein coding genes.

## Species taxonomy

Eukaryota; Metazoa; Ecdysozoa; Arthropoda; Hexapoda; Insecta; Pterygota; Neoptera; Endopterygota; Hymenoptera; Tenthredinoidea; Athaliidae; *Athalia*; *Athalia rosae* (Linnaeus, 1758) (NCBI:txid37344).

## Background

*Athalia rosae* is a small (6 to 8 mm), orange sawfly marked with black on the head, antennae, mesonotum, tibiae, and tarsi. It is commonly referred to in Britain as the Turnip Sawfly, a reference to its importance as a pest of turnip, mustard, and other crops throughout temperate and subtropical zones of Europe, Asia, and Africa. In Britain, it was a serious pest during the 18th and early 19th century before almost disappearing until the 1940s, since when it has become common in England and Wales and occurring in smaller numbers throughout Scotland ( Benson, 1952). The species breeds in Britain, but its numbers are boosted by large migrations from the near continent. Adults are on the wing from April to October. *A. rosae* is easily distinguished from other *Athalia* species from the presence of a distinctive orange and black chequerboard pattern on the mesonotum ( Benson, 1952). Conversely, the dark blue-grey larvae can be difficult to separate from other species within the genus. The larvae feed on a broad range of brassicas including *Armoracia rusticana* (Horseradish), *Barbarea vulgaris* (Winter-cress), *Brassica juncea* (Chinese Mustard), *Brassica napus* (Rape), *Brassica nigra* (Black Mustard), and *Brassica rapa* (Turnip) ( Liston, 1995). The species is multivoltine.

*Athalia* has for a long time been recognised as an ancient and distinct genus within the Tenthredinidae ( Cameron, 1882). Recent research comparing sawfly mitochondrial genomes ( Niu *et al.*, 2022) has concluded that the genus *Athalia* is within a newly defined family, Athaliidae, separate from the Tenthredinidae. The chromosomal genome of *Athalia rosae* will help with research into the diversification and contribute insight into phylogeographic distribution pattern of this group of sawflies across their Eurasian and African range ( Niu *et al.*, 2022).

### Genome sequence report

**Figure 1.**
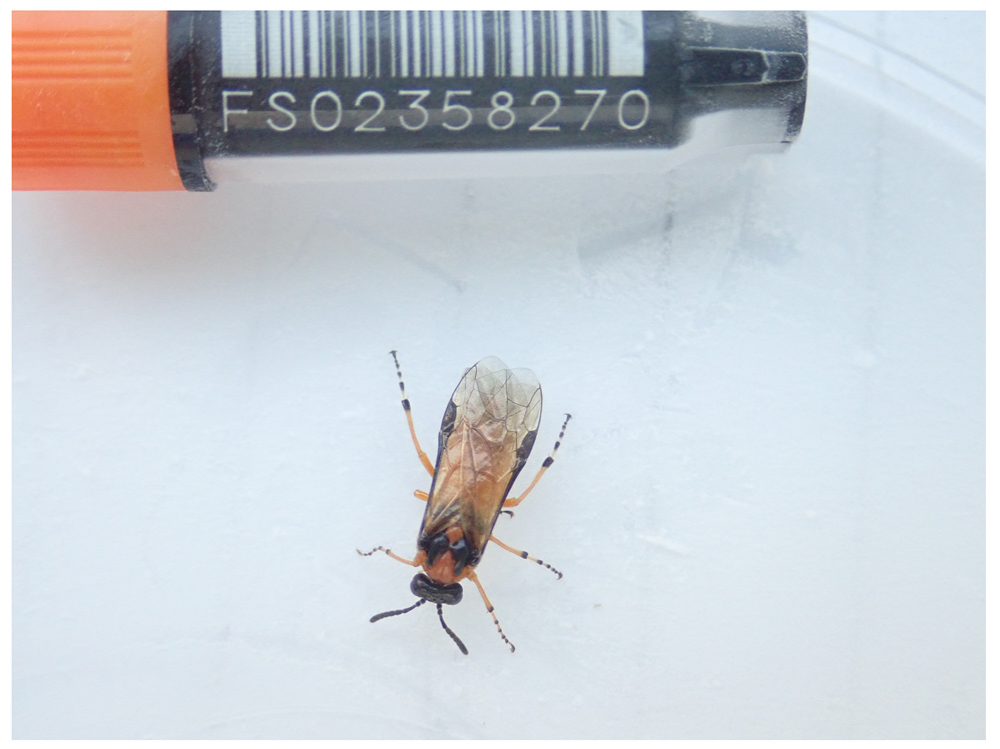
Photograph of the *Athalia rosae* (iyAthRosa1) specimen used for genome sequencing.

The genome was sequenced from one female *Athalia rosae* ( Figure 1) collected from Wytham Woods, UK (latitude 51.77, longitude –1.34). A total of 103-fold coverage in Pacific Biosciences single-molecule HiFi long reads and 205-fold coverage in 10X Genomics read clouds were generated. Primary assembly contigs were scaffolded with chromosome conformation Hi-C data. Manual assembly curation corrected 39 missing or mis-joins and removed five haplotypic duplications, reducing the scaffold number by 72.55%, and increasing the scaffold N50 by 90%.

**Figure 2.**
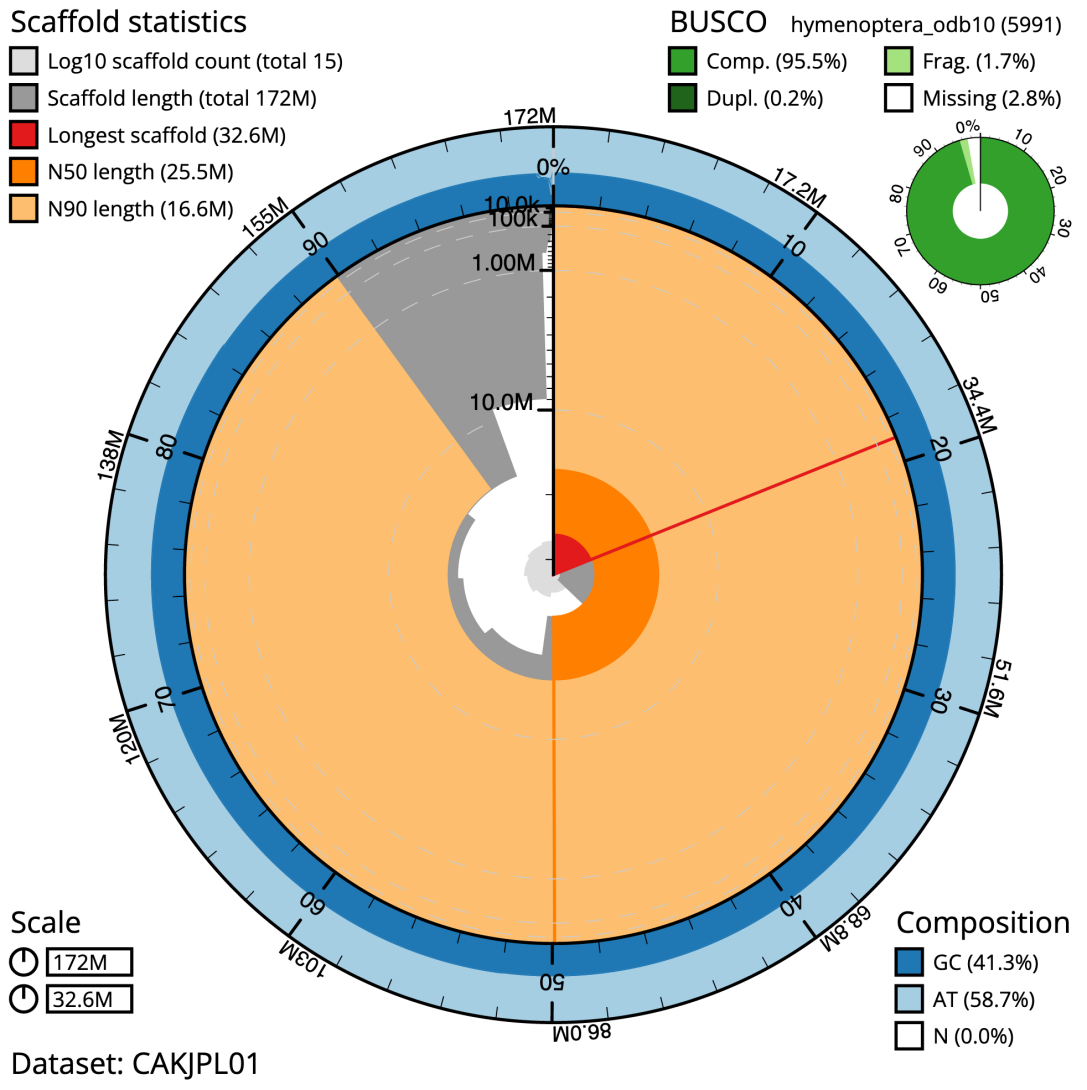
Genome assembly of *Athalia rosae*, iyAthRosa1.1: metrics. The BlobToolKit Snailplot shows N50 metrics and BUSCO gene completeness. The main plot is divided into 1,000 size-ordered bins around the circumference with each bin representing 0.1% of the 171,971,399 bp assembly. The distribution of scaffold lengths is shown in dark grey with the plot radius scaled to the longest scaffold present in the assembly (32,626,317 bp, shown in red). Orange and pale-orange arcs show the N50 and N90 scaffold lengths (25,530,711 and 16,594,773 bp), respectively. The pale grey spiral shows the cumulative scaffold count on a log scale with white scale lines showing successive orders of magnitude. The blue and pale-blue area around the outside of the plot shows the distribution of GC, AT and N percentages in the same bins as the inner plot. A summary of complete, fragmented, duplicated and missing BUSCO genes in the hymenoptera_odb10 set is shown in the top right. An interactive version of this figure is available at https://blobtoolkit.genomehubs.org/view/iyAthRosa1.1/dataset/CAKJPL01/snail.

The final assembly has a total length of 172.0 Mb in 14 sequence scaffolds with a scaffold N50 of 25.5 Mb ( Table 1). Most (99.4%) of the assembly sequence was assigned to eight chromosomal-level scaffolds. Chromosome-scale scaffolds confirmed by the Hi-C data are named in order of size ( Figure 2– Figure 5; Table 2). The assembly has a BUSCO v5.3.2 ( Manni *et al.*, 2021) completeness of 95.5% (single 95.3%, duplicated 0.2%) using the hymenoptera_odb10 reference set. While not fully phased, the assembly deposited is of one haplotype. Contigs corresponding to the second haplotype have also been deposited.

**Table 1.** Genome data for *Athalia rosae*, iyAthRosa1.1.

Project accession data		
Assembly identifier	iyAthRosa1.1	
Species	*Athalia rosae*	
Specimen	iyAthRosa1	
NCBI taxonomy ID	37344	
BioProject	PRJEB45200	
BioSample ID	SAMEA7520481	
Isolate information	iyAthRosa1 (PacBio, Chromium and Hi-C) iyAthRosa6 (RNA-Seq)	
Assembly metrics *		*Benchmark*
Consensus quality (QV)	50.2	*≥ 50*
*k*-mer completeness	99.97	*≥ 95%*
BUSCO **	C:95.5%[S:95.3%,D:0.2%], F:1.7%,M:2.8%,n:5,991	*C ≥ 95%*
Percentage of assembly mapped to chromosomes	99.4%	*≥ 95%*
Sex chromosomes	N/A	*localised homologous pairs*
Organelles	Mitochondrial genome assembled	*complete single alleles*
Raw data accessions		
PacificBiosciences SEQUEL II	ERR6548410	
10X Genomics Illumina	ERR6054977–ERR6054980	
Hi-C Illumina	ERR6054981	
PolyA RNA-Seq Illumina	ERR9434987	
Genome assembly		
Assembly accession	GCA_917208135.1	
*Accession of alternate haplotype*	GCA_917207985.2	
Span (Mb)	172.0	
Number of contigs	71	
Contig N50 length (Mb)	7.1	
Number of scaffolds	14	
Scaffold N50 length (Mb)	25.5	
Longest scaffold (Mb)	32.6	
**Genome annotation**		
Number of protein-coding genes	11,393	
Average length of coding sequence (bp)	8,560.96	
Average number of exons per transcript	6.78	
Average number of introns per transcript	5.78	
Average intron size (bp)	916.89	

* Assembly metric benchmarks are adapted from column VGP-2020 of “Table 1: Proposed standards and metrics for defining genome assembly quality” from ( Rhie *et al.*, 2021). ** BUSCO scores based on the hymenoptera_odb10 BUSCO set using v5.3.2. C = complete [S = single copy, D = duplicated], F = fragmented, M = missing, n = number of orthologues in comparison. A full set of BUSCO scores is available at https://blobtoolkit.genomehubs.org/view/iyAthRosa1.1/dataset/CAKJPL01/busco.

**Figure 3.**
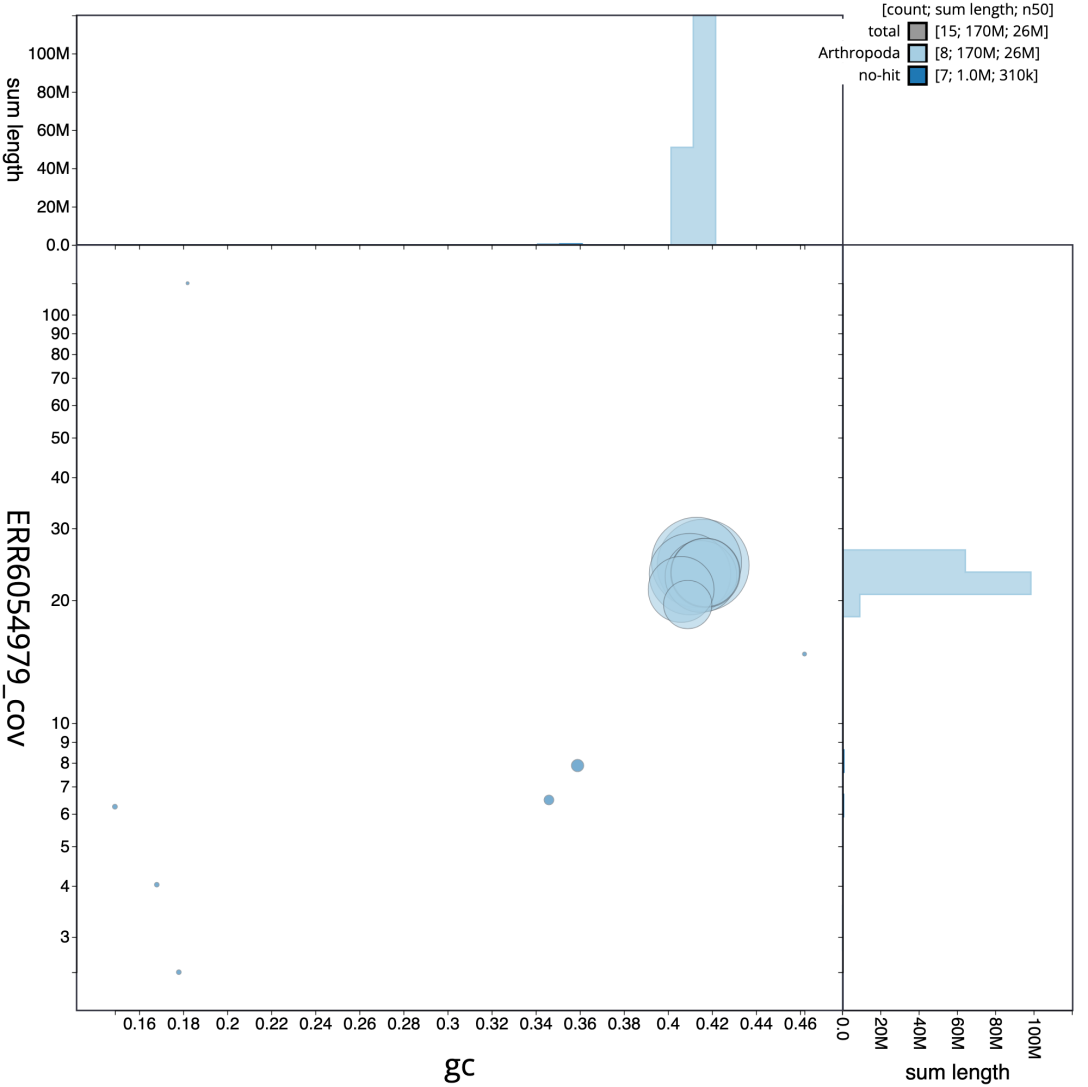
Genome assembly of *Athalia rosae*, iyAthRosa1.1: GC coverage. BlobToolKit GC-coverage plot. Scaffolds are coloured by phylum. Circles are sized in proportion to scaffold length. Histograms show the distribution of scaffold length sum along each axis. An interactive version of this figure is available at https://blobtoolkit.genomehubs.org/view/iyAthRosa1.1/dataset/CAKJPL01/blob.

**Figure 4.**
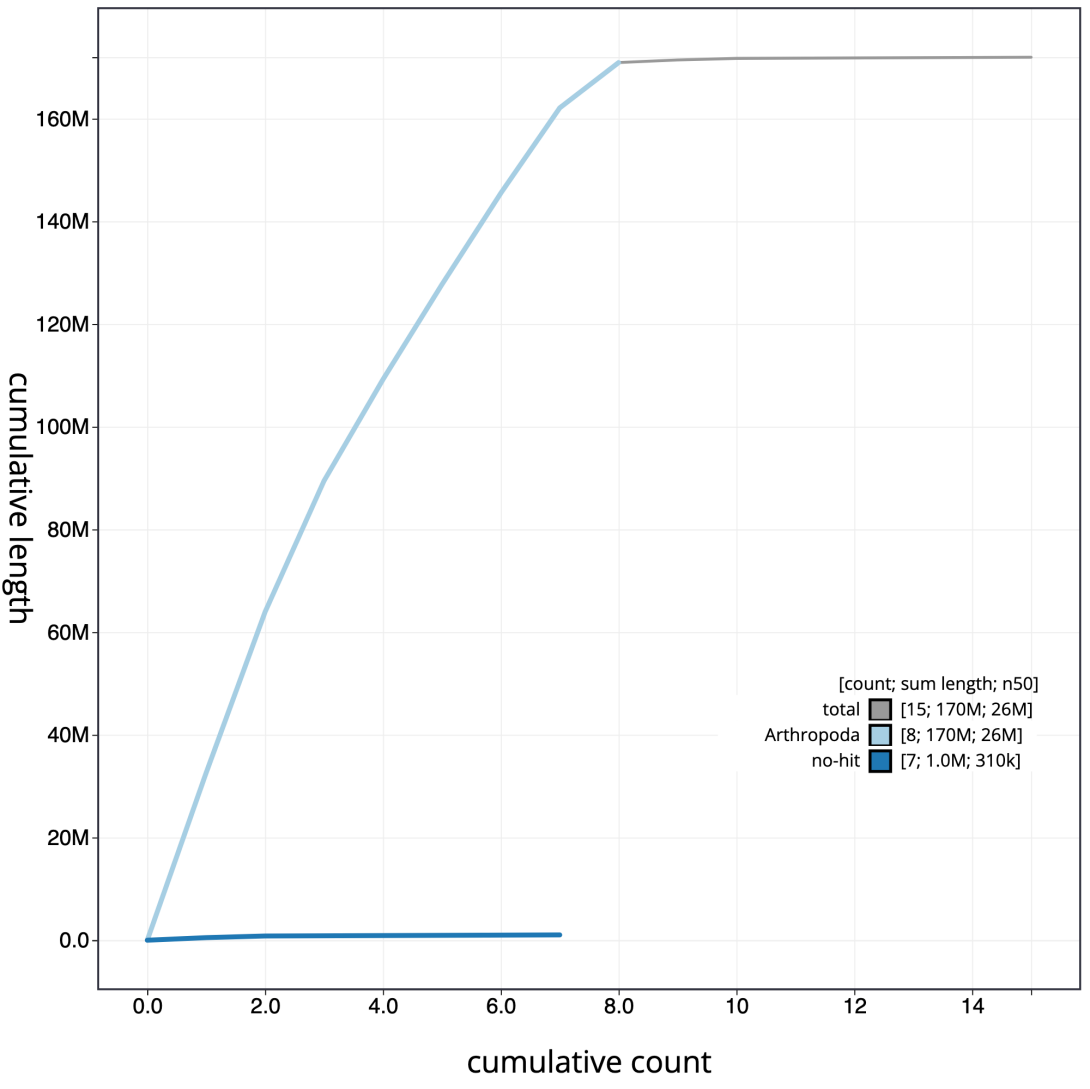
Genome assembly of *Athalia rosae*, iyAthRosa1.1: cumulative sequence. BlobToolKit cumulative sequence plot. The grey line shows cumulative length for all scaffolds. Coloured lines show cumulative lengths of scaffolds assigned to each phylum using the buscogenes taxrule. An interactive version of this figure is available at https://blobtoolkit.genomehubs.org/view/iyAthRosa1.1/dataset/CAKJPL01/cumulative.

**Figure 5.**
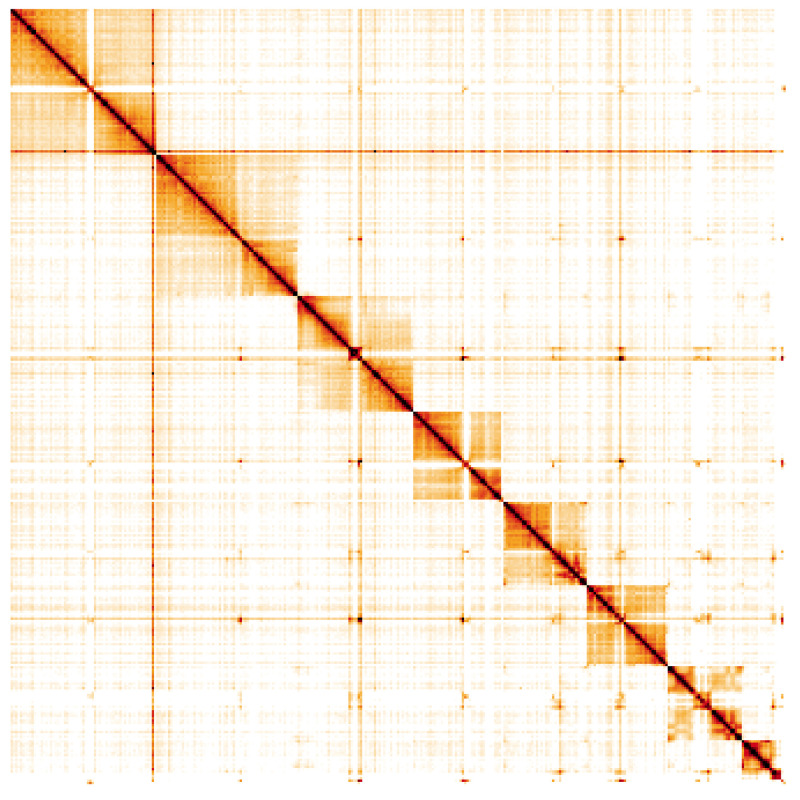
Genome assembly of *Athalia rosae*, iyAthRosa1.1: Hi-C contact map. Hi-C contact map of the iyAthRosa1.1 assembly, visualised using HiGlass. Chromosomes are shown in order of size from left to right and top to bottom. An interactive version of this figure may be viewed at https://genome-note-higlass.tol.sanger.ac.uk/l/?d=QqOObBEaRcG_q4y7QCiq1Q.

**Table 2.** Chromosomal pseudomolecules in the genome assembly of *Athalia rosae*, iyAthRosa1.

INSDC accession	Chromosome	Size (Mb)	GC%
OU795000.1	1	32.63	41.6
OU795001.1	2	31.32	41.3
OU795002.1	3	25.53	41
OU795003.1	4	19.73	41.5
OU795004.1	5	18.48	41.7
OU795005.1	6	17.81	41.7
OU795006.1	7	16.59	40.6
OU795007.1	8	8.84	40.9
OU795008.1	MT	0.02	18.1
-	unplaced	1.02	32.6

### Genome annotation report

Annotation of the GCA_917208135.1 assembly was generated using the Ensembl genome annotation pipeline ( Table 1; https://rapid.ensembl.org/Athalia_rosae_GCA_917208135.1/). The resulting annotation includes 11,393 protein coding genes with an average length of 8,560.96 and an average coding length of 1,651.72, and 2,142 non-protein coding genes. There is an average of 6.78 exons and 5.78 introns per canonical protein coding transcript, with an average intron length of 916.89.

## Methods

### Sample acquisition and nucleic acid extraction

One *Athalia rosae* (iyAthRosa1) specimen was collected by netting in Wytham Woods, Oxfordshire (biological vice-county: Berkshire), UK (latitude 51.77, longitude –1.34) on 28 August 2019. The specimen was collected and identified by Liam Crowley (University of Oxford) and snap-frozen on dry ice. This specimen was used for DNA sequencing.

A second specimen (iyAthRosa6) was collected using by netting in Hartslock Reserve, Oxfordshire, UK (latitude 51.51, longitude –1.11) on 20 August 2020. This specimen was collected and identified by Gavin Broad (Natural History Museum) and snap-frozen on dry ice. This specimen was used for RNA-Seq (iyAthRosa6).

DNA was extracted at the Tree of Life laboratory, Wellcome Sanger Institute (WSI). The iyAthRosa1 sample was weighed and dissected on dry ice with tissue set aside for Hi-C sequencing. Thorax tissue was disrupted using a Nippi Powermasher fitted with a BioMasher pestle. High molecular weight (HMW) DNA was extracted using the Qiagen MagAttract HMW DNA extraction kit. Low molecular weight DNA was removed from a 20 ng aliquot of extracted DNA using 0.8X AMpure XP purification kit prior to 10X Chromium sequencing; a minimum of 50 ng DNA was submitted for 10X sequencing. HMW DNA was sheared into an average fragment size of 12–20 kb in a Megaruptor 3 system with speed setting 30. Sheared DNA was purified by solid-phase reversible immobilisation using AMPure PB beads with a 1.8X ratio of beads to sample to remove the shorter fragments and concentrate the DNA sample. The concentration of the sheared and purified DNA was assessed using a Nanodrop spectrophotometer and Qubit Fluorometer and Qubit dsDNA High Sensitivity Assay kit. Fragment size distribution was evaluated by running the sample on the FemtoPulse system.

RNA was extracted from whole organism tissue of iyAthRosa6 in the Tree of Life Laboratory at the WSI using TRIzol, according to the manufacturer’s instructions. RNA was then eluted in 50 μl RNAse-free water and its concentration assessed using a Nanodrop spectrophotometer and Qubit Fluorometer using the Qubit RNA Broad-Range (BR) Assay kit. Analysis of the integrity of the RNA was done using Agilent RNA 6000 Pico Kit and Eukaryotic Total RNA assay.

### Sequencing

Pacific Biosciences HiFi circular consensus and 10X Genomics read cloud DNA sequencing libraries were constructed according to the manufacturers’ instructions. Poly(A) RNA-Seq libraries were constructed using the NEB Ultra II RNA Library Prep kit. DNA and RNA sequencing were performed by the Scientific Operations core at the WSI on Pacific Biosciences SEQUEL II (HiFi), Illumina HiSeq 4000 (RNA-Seq) and HiSeq X Ten (10X) instruments. Hi-C data were also generated from head and thorax tissue of iyAthRosa1 using the Arima v2 kit and sequenced on the Illumina NovaSeq 6000 instrument.

### Genome assembly

Assembly was carried out with Hifiasm ( Cheng *et al.*, 2021) and haplotypic duplication was identified and removed with purge_dups ( Guan *et al.*, 2020). One round of polishing was performed by aligning 10X Genomics read data to the assembly with Long Ranger ALIGN, calling variants with freebayes ( Garrison & Marth, 2012). The assembly was then scaffolded with Hi-C data ( Rao *et al.*, 2014) using SALSA2 ( Ghurye *et al.*, 2019). The assembly was checked for contamination and corrected using the gEVAL system ( Chow *et al.*, 2016) as described previously ( Howe *et al.*, 2021). Manual curation was performed using gEVAL, HiGlass ( Kerpedjiev *et al.*, 2018) and Pretext ( Harry, 2022). The mitochondrial genome was assembled using MitoHiFi ( Uliano-Silva *et al.*, 2022), which performed annotation using MitoFinder ( Allio *et al.*, 2020). The genome was analysed and BUSCO scores generated within the BlobToolKit environment ( Challis *et al.*, 2020). Table 3 contains a list of all software tool versions used, where appropriate.

**Table 3.** Software tools and versions used.

Software tool	Version	Source
BlobToolKit	3.5.0	Challis *et al.*, 2020
freebayes	1.3.1-17- gaa2ace8	Garrison & Marth, 2012
gEVAL	N/A	Chow *et al.*, 2016
Hifiasm	0.15.1	Cheng *et al.*, 2021
HiGlass	1.11.6	Kerpedjiev *et al.*, 2018
Long Ranger ALIGN	2.2.2	https://support.10xgenomics.com/genome-exome/ software/pipelines/latest/advanced/other-pipelines
MitoHiFi	2	Uliano-Silva *et al.*, 2022
PretextView	0.2	Harry, 2022
purge_dups	1.2.3	Guan *et al.*, 2020
SALSA	2.2	Ghurye *et al.*, 2019

### Genome annotation

The Ensembl gene annotation system ( Aken *et al.*, 2016) was used to generate annotation for the *A. rosae* assembly (GCA_917208135.1). Annotation was created primarily through alignment of transcriptomic data to the genome, with gap filling via protein to-genome alignments of a select set of proteins from UniProt ( UniProt Consortium, 2019).

### Ethics and compliance issues

The materials that have contributed to this genome note have been supplied by a Darwin Tree of Life Partner. The submission of materials by a Darwin Tree of Life Partner is subject to the Darwin Tree of Life Project Sampling Code of Practice. By agreeing with and signing up to the Sampling Code of Practice, the Darwin Tree of Life Partner agrees they will meet the legal and ethical requirements and standards set out within this document in respect of all samples acquired for, and supplied to, the Darwin Tree of Life Project. All efforts are undertaken to minimise the suffering of animals used for sequencing. Each transfer of samples is further undertaken according to a Research Collaboration Agreement or Material Transfer Agreement entered into by the Darwin Tree of Life Partner, Genome Research Limited (operating as the Wellcome Sanger Institute), and in some circumstances other Darwin Tree of Life collaborators.

## Data Availability

European Nucleotide Archive: *Athalia rosae* (turnip sawfly). Accession number PRJEB45200; https://identifiers.org/ena.embl/PRJEB45200. ( Wellcome Sanger Institute, 2021) The genome sequence is released openly for reuse. The *Athalia rosae* genome sequencing initiative is part of the Darwin Tree of Life (DToL) project. All raw sequence data and the assembly have been deposited in INSDC databases. Raw data and assembly accession identifiers are reported in Table 1.

## Author information

Members of the University of Oxford and Wytham Woods Genome Acquisition Lab are listed here: https://doi.org/10.5281/zenodo.4789928.

Members of the Natural History Museum Genome Acquisition Lab are listed here: https://doi.org/10.5281/zenodo.4790043.

Members of the Darwin Tree of Life Barcoding collective are listed here: https://doi.org/10.5281/zenodo.4893703.

Members of the Wellcome Sanger Institute Tree of Life programme are listed here: https://doi.org/10.5281/zenodo.4783585.

Members of Wellcome Sanger Institute Scientific Operations: DNA Pipelines collective are listed here: https://doi.org/10.5281/zenodo.4790455.

Members of the Tree of Life Core Informatics collective are listed here: https://doi.org/10.5281/zenodo.5013541.

Members of the Darwin Tree of Life Consortium are listed here: https://doi.org/10.5281/zenodo.4783558.
