# Supplementary material for: The genome sequence of the Turnip Sawfly, Athalia rosae (Linnaeus, 1758)
Source: Wellcome Open Res. 2023 Feb 17;8:87. [Version 1] doi: 10.12688/wellcomeopenres.18993.1 (PMC10792271; doi:10.12688/wellcomeopenres.18993.1)
Supplement: Peer review history

# Reviewer report: Reviewer response for version 1

**DOI:** 10.21956/wellcomeopenres.21057.r72240

**Published:** 2024-01-31

**Authors:** Rodrigo Pracana

**Affiliations:** 1 Queen Mary University of London, London, UK

The authors provide a genome assembly and annotation for the Turnip Sawfly ( *Athalia rosae*). Like all assemblies provided by the Darwin Tree of Life Consortium, this dataset is an important resource in itself. Furthermore, it will prove very useful for people interested in Hymenopteran diversity and evolution. Despite my comment below, I trust the assembly provided and I thank the authors for the huge amount of effort put in to create this resource.

 I find that the level of detail provided for the sample acquisition, extraction and sequencing is thorough. However, this manuscript does not reference in enough detail the bioinformatic methods used to allow replication by others. For example, it is unclear whether the sequenced reads were preprocessed, and the type of parameters used in the different steps reported. Of course, it is not in the scope of this manuscript to provide a particularly detailed view of bioinformatic analyses performed by DToL, but I would expect DToL to publish an explanation of its bioinformatic pipelines for individual manuscripts to reference.

 Minor points:

 It should be made explicit that BUSCO was made using the genome mode (not the transcriptome or protein mode), so that it is unambiguous that the completeness values reported do reflect the quality of the assembly and not the quality of the annotation. (There would be value in reporting the completeness of the annotation, but this is not essential for a data report).

 "average length of 8,560.96 and an average coding length of 1,651.72". I would include a unit (nucleotides) and explicitly say whether or not the length of 8,560.96 includes introns or not.

 "but its numbers are boosted by large migrations from the near continent". Using the term "mainland Europe" is more clear that "the near continent".

 What is dataset CAKJPL01 referenced in Fig. 2?

Are sufficient details of methods and materials provided to allow replication by others?

Partly

Is the rationale for creating the dataset(s) clearly described?

Yes

Are the datasets clearly presented in a useable and accessible format?

Yes

Are the protocols appropriate and is the work technically sound?

Yes

Reviewer Expertise:

Hymenopteran genomics, comparative genomics

I confirm that I have read this submission and believe that I have an appropriate level of expertise to confirm that it is of an acceptable scientific standard, however I have significant reservations, as outlined above.

# Reviewer report: Reviewer response for version 1

**DOI:** 10.21956/wellcomeopenres.21057.r72236

**Published:** 2024-01-23

**Authors:** Shu-Jun Wei

**Affiliations:** 1 Beijing Academy of Agriculture and Forestry Sciences, Beijing, China

This study reported the chromosome-level genome for the turnip sawfly (Hymenoptera; Athaliidae) using Pacific Biosciences single-molecule HiFi long reads, 10X Genomics, and Hi-C technologies. This genome assembly has a BUSCO completeness of 95.5%. The results will help understand the phylogeographic distribution of sawflies and the evolution of Hymenoptera. Here are some points for clarification:

1. Are there any voucher specimens or DNA available?
2. Can you provide information about the completeness of the annotated protein-coding genes?
3. Was the mitochondrial genome annotated? Please include the annotation results for the mitochondrial genome.

Are sufficient details of methods and materials provided to allow replication by others?

Yes

Is the rationale for creating the dataset(s) clearly described?

Yes

Are the datasets clearly presented in a useable and accessible format?

Yes

Are the protocols appropriate and is the work technically sound?

Yes

Reviewer Expertise:

Population genetics, genomics, and pest control.

I confirm that I have read this submission and believe that I have an appropriate level of expertise to confirm that it is of an acceptable scientific standard.

# Reviewer report: Reviewer response for version 1

**DOI:** 10.21956/wellcomeopenres.21057.r72239

**Published:** 2024-01-16

**Authors:** Xiaohan Shu

**Affiliations:** 1 Zhejiang University, Sanya, China

This article describes the genome sequencing and annotation of the Turnip Sawfly ( *Athalia rosae*). The genome of *Athalia rosae* is a good resource and useful for researchers studying sawfly. Overall this genome is high quality and the manuscript provides a sufficient description of the methods used to generate the genome.

Are sufficient details of methods and materials provided to allow replication by others?

Yes

Is the rationale for creating the dataset(s) clearly described?

Yes

Are the datasets clearly presented in a useable and accessible format?

Yes

Are the protocols appropriate and is the work technically sound?

Yes

Reviewer Expertise:

insect genomics, ecology & evolution

I confirm that I have read this submission and believe that I have an appropriate level of expertise to confirm that it is of an acceptable scientific standard.

# Reviewer report: Reviewer response for version 1

**DOI:** 10.21956/wellcomeopenres.21057.r58697

**Published:** 2023-07-14

**Authors:** Sandro Patroca da Silva

**Affiliations:** 1 Evandro Chagas Institute, Ananindeua, Brazil

The article entitled "The genome sequence of the Turnip Sawfly, Athalia rosae (Linnaeus, 1758)" describes the sequencing of an insect species using different methodologies and sequencing platforms, as well as bioinformatics methodologies to obtain the nuclear and mitochondrial genome of the Turnip Sawfly. In my opinion, the laboratory methodologies and computational analysis tools are appropriate, and I did not observe any issues with their utilization.

Are sufficient details of methods and materials provided to allow replication by others?

Yes

Is the rationale for creating the dataset(s) clearly described?

Yes

Are the datasets clearly presented in a useable and accessible format?

Yes

Are the protocols appropriate and is the work technically sound?

Yes

Reviewer Expertise:

I am a virologist and have expertise in NGS technology and bioinformatic analysis. At the moment, I am beginning to work with the mitochondrial genome of arthropods.

I confirm that I have read this submission and believe that I have an appropriate level of expertise to confirm that it is of an acceptable scientific standard.
